# Reduced Venous Compliance in Young Women with Type 1 Diabetes – Further Aggravated by Prolonged Elevated Levels of HbA1c

**DOI:** 10.3389/fendo.2016.00126

**Published:** 2016-09-21

**Authors:** Marcus Lindenberger

**Affiliations:** ^1^Department of Cardiology and Department of Medical and Health Sciences, Linköping University, Linköping, Sweden

**Keywords:** type 1 diabetes, venous compliance, HbA1c, hemodynamics, retinopathy, women

## Abstract

**Background:**

Young patients with diabetes present with reduced compensatory responses to hypovolemic stress. Less compliant veins could be a contributing factor, since roughly two-thirds of the blood volume resides in the venous system as a blood reservoir, adjusting proper venous inflow to the heart. The aim of this study was to measure venous compliance and lower limb blood pooling during hypovolemic stress, and to correlate them to indices of diabetes severity and glucose control.

**Methods:**

Fifteen young women with type 1 diabetes (DW) and 18 healthy age-matched women (C) were subjected to lower body negative pressure (LBNP) (11–44 mmHg), creating hypovolemic stress. Lower limb blood pooling was measured with strain gage technique and venous compliance calculated as the relationship between ∆V/∆P.

**Results:**

DW presented with reduced blood pooling (e.g., blood pooling during LBNP of 44 mmHg, DW, 1.69 ± 0.10; C, 2.10 ± 0.08 (ml/100 ml), and *P* = 0.003). Calculated venous compliance was also reduced in DW (e.g., compliance at 20 mmHg, DW, 0.046 ± 0.003; C, 0.059 ± 0.002 (ml/100 ml/mmHg), and *P* = 0.002). A progressive reduction in both venous compliance (*P* < 0.007) and blood pooling (*P* < 0.005) was seen with increasing level of HbA_1c_, and furthermore, less strongly associated with presence of microvascular disease (signs of retinopathy).

**Conclusion:**

Women with type 1 diabetes present with both reduced venous compliance and blood pooling. The reductions were particularly present in patients with long-standing poor glycemic control.

## Introduction

Orthostatic hypotension is more common in people with type 1 diabetes (1–4). Diabetes is also associated with hemodynamic instability and reduced tolerance to rapidly induced hypovolemia (e.g., during anesthesia) (1, 2, 5) and prolonged hypovolemia (e.g., hemodialysis) (6), aggravated with the presence of cardiovascular autonomic neuropathy (CAN) (2, 4, 5). Nevertheless, diabetes patients without signs of or only mild CAN are also at risk of hypotension during orthostatic stress and anesthesia, pointing toward other causal factors involved (1, 5).

Lower body negative pressure (LBNP) is a well-proven technique to mimic orthostatic and central hypovolemic stress by pooling blood in the lower part of the body (7). Reduced lower limb blood pooling has previously been noted in men with type 1 diabetes, further associated with reduced blood pooling in men with detectable microvascular disease (8). Reduced venous compliance has been noted in subjects with diabetes (9, 10), but not studied in conjunction with orthostatic stress. Reduced venous compliance could be beneficial during orthostatic stress, reducing the amount of blood pooled in the lower limbs (11). However, reduced venous compliance could also be detrimental. High vessel wall compliance is mandatory for efficient mobilization of peripheral venous capacitance blood to the central circulation to uphold venous return, cardiac output, and blood pressure during an orthostatic challenge (12, 13), We have recently presented reduced mobilization of peripheral capacitance blood in both men and women with type 1 diabetes (8, 14), aggravated with the severity of the disease, i.e., presence of microvascular disease (8) and level of Hb_A1c_ (14). Female gender seems especially predisposed to diabetes-associated reduction in compliance with major elastic arteries (15). Furthermore, young healthy women have lower tolerance to orthostatic stress than men (16). Reduced speed of initial blood pooling has been linked with orthostatic intolerance in women (17). Collectively, this indicates young women with diabetes as a particularly interesting group to study.

The aim of this present study was to assess venous compliance and lower limb blood pooling in healthy women and in young women with type 1 diabetes and, furthermore, to correlate venous compliance with known risk factors associated with type 1 diabetes (e.g., age of diabetes onset, diabetes duration, presence of microvascular disease, and level of glycated hemoglobin). We hypothesized that venous compliance and blood pooling would be reduced in women with type 1 diabetes, and that the reductions would be associated with markers of diabetes severity.

## Materials and Methods

### Participants

Women with type 1 diabetes enrolled as outdoor patients at the Department of Endocrinology at Linkoping University Hospital were asked to participate in the study if they met the inclusion and exclusion criteria. The inclusion criteria were: age between 18 and 30 years, duration of diabetes of at least 5 years, and willingness to participate in the study. Exclusion criteria: current smoking, sedentary lifestyle/obesity, and cardiovascular disease. Fifteen women with type 1 diabetes (DW) were included in the study. They were all on a multiple-dose insulin regime or treated with insulin pump without any other chronic cardiovascular medication. HbA1c was analyzed with Mono *S*-technique and then calculated to IFCC values using the formula: HbA1c IFCC (mmol/mol) = 10.45 × [HbA1c, Mono S %] – 10.62. HbA_1c_ in DW were 66 ± 2 mmol/mol (range 47–82 mmol/mol), reference value <45 mmol/mol, with background data in DW presented in Table 1. Eighteen healthy age-matched women (C) were selected from the general population after public advertising meeting the same inclusion/exclusion criteria as DW with the exception for type 1 diabetes. See Table 2 for cardiovascular data. A recent study focusing on cardiovascular compensatory responses to hypovolemic stress (including mobilization of peripheral blood to central circulation and net fluid absorption of extravascular fluid) included all DW and 16 controls (14). Two additional healthy women were included in the present study, previously excluded from (14) due to missing key measurements in homeostatic regulation. As such, the present study and recent study (14) share basal resting cardiovascular parameters. On the other hand, venous compliance has not previously been studied in our research group in either men or women with type 1 diabetes. Subgroup analyses were conducted for differences in glycemic control, the presence of microvascular disease, and other parameters presented in Table 1. In order to elucidate the effect of hyperglycemia over time, mean HbA_1c_ from 5 years preceding the study was calculated [HbA_1c5_, in analogy with (14, 18)] being 67 ± 2 mmol/mol (range 51–84 mmol/mol) in DW, while HbA_1c_ in C (*n* = 8) was (31 ± 1 mmol/mol, range 26–33 mmol/mol, measured once). Based on their HbA_1c_, all participating women were divided into four subgroups: normal (*n* = 18) (HbA_1c5_ < 45 mmol/mol); mild (*n* = 5) [HbA_1c5_ range 51–60 mmol/mol (mean 56 ± 2 mmol/mol)]; moderate (*n* = 5) [HbA_1c5_ range 61–70 mmol/mol (mean 67 ± 2 mmol/mol)]; poor (*n* = 5) [HbA_1c5_ range > 70 mmol/mol (mean 77 ± 2 mmol/mol)] (overall subgroup reduction in HbA_1c5_, *P* < 0.0001; mild vs. moderate, *P* = 0.005 and poor, *P* = 0.0005; moderate vs. poor *P* = 0.01). Microvascular disease was present in seven DW (all diagnosed with background retinopathy of whom four with minimal or slight background retinopathy), comprising the subgroup of RET+, while the remaining eight DW without signs of retinopathy formed RET−. All women were scheduled in the middle part of the menstrual cycle, with 9 C and 6 DW on oral contraceptives. No impact of menstrual cycle or oral contraceptives have been seen on venous compliance (12, 19). Each subject provided written informed consent to the experiments approved by the local Ethics Committee of Linkoping University and conformed to the Declaration of Helsinki.

**Table 1:** **Characteristics of women with type 1 diabetes**.

Parameter	Mean + SE	Range
Age at onset of diabetes, years	11 ± 1	1–19
Duration of diabetes, years	13 ± 1	5–23
HbA_1c_ at study, mmol/mol	66 ± 2	47–82
Mean HbA_1c5_, mmol/mol	67 ± 2	51–84
Hemoglobin, g/dl	13.8 ± 0.1	12.8–14.9
GRF, ml/min/1.73 m^2^	93 ± 3	71–124
Total cholesterol, mmol/l	4.3 ± 0.2	3.4–6.2
Albuminuria, mg/mmol	6 ± 2	0–16
Total insulin dosage/day, IE	49 ± 4	25–82
Insulin dosage/body weight, E/kg	0.73 ± 0.05	0.35–1.16
Blood glucose level, mmol/l	8.7 ± 1	4.2–12.8
Retinopathy, background	7	
Retinopathy, proliferative	0	
Neuropathy	0	
Nephropathy	0	

**Table 2:** **Cardiovascular parameters in DW and C**.

Parameter	DW	C	*P*-value
*N*	15	18	
HR, beats/min	69 ± 2	57 ± 1	**0.0001**
SBP, mmHg	110 ± 2	105 ± 1	0.06
DBP, mmHg	61 ± 1	65 ± 1	**0.04**
MAP, mmHg	78 ± 1	78 ± 1	0.98
PP, mmHg	49 ± 2	41 ± 2	**0.001**
FBF, ml/100 ml/min	2.6 ± 0.2	1.9 ± 0.2	**0.01**
FVR, units	31 ± 2	45 ± 3	**0.003**
FVC, units (E^−3^)	34 ± 2	25 ± 2	**0.02**
P-NE, pmol/l	1.2 ± 0.2	1.2 ± 0.1	0.70

*HR, heart rate; SBP, systolic blood pressure; DBP, diastolic blood pressure; MAP, mean arterial pressure; PP, pulse pressure; FBF, forearm blood pressure; FVR, forearm vascular resistance; FVC, forearm vascular conductance; P-NE, plasma norepinephrine*.

*Bold expresses significant group differences*.

### Lower Body Negative Pressure

The experiments started 1 h after a light meal randomly in the morning or afternoon. No circadian variations have been seen in previous, similar experiments in our lab (12). Room temperature was held constant between 23 and 25°C to avoid the subjects to get chilled. The subjects were instructed to abstain from caffeine on the day of investigation. DW were instructed to take their ordinary insulin doses prior to the experiment. The subjects were placed in the supine position with the lower part of the body up to the level of the iliac crest enclosed in an airtight box connected to a vacuum source, enabling stable negative pressure to be produced within 5 s (LBNP), continuously measured and held constant by a rheostat. During LBNP, 80% of the negative pressure is transmitted to the underlying muscle tissue of the leg irrespective of muscle depth, time, and magnitude, leading to a defined increase in transmural pressure over the vessel wall, with a concomitant vessel dilatation and blood pooling (11).

### Lower Limb Blood Pooling

To measure the amount of blood pooling evoked by LBNP, calf volume changes (ml/100 ml) were measured with mercury-in-silicone strain gage plethysmography applied at the maximal circumference of the right calf. To avoid any confounding external tissue pressure, the lowest part of the calf was 2 cm above the floor of the LBNP chamber. The subjects rested in the supine position for at least 30 min to ensure stable calf volume and arterial inflow prior to the start of experiments. Interindividual supine resting venous pressure has been shown to be fairly constant (20).

Momentarily after LBNP initiation, a rapid increase in calf volume is seen (blood pooling) followed by a slower, but continuous rise caused by net capillary fluid filtration from blood to surrounding tissue. At cessation, there is a rapid decrease in calf volume corresponding with the increase at onset of LBNP (21). Fully developed blood pooling is achieved well within 3 min at LBNP levels used in the present study, and net capillary fluid filtration continually and linearly increases calf volume thereafter (21). The blood pooling was, therefore, calculated from calf volume at baseline to the line defined from the filtration slope (14, 21). The reproducibility of venous blood pooling measurements with this approach is good (CV <10%) (22). We also assessed calf volume increase (every 5 s) and calculated mean rate of blood pooling (ml/100 ml/min) during the first min of LBNP.

After at least 30 min of complete rest in the supine position, LBNP of 11 mmHg was applied for 4–6 min followed by complete rest for 5–10 min. LBNP of 22 and 44 mmHg was then applied in similar fashion with rest in between. Continuous recordings of calf volume ensured that basal calf volume was restored and stable before each LBNP session. Continuous, online recordings confirmed a clear and stable filtration slope for at least 2 min used to separate blood pooling from net fluid filtration. The above protocol was in the majority of cases repeated, and the mean blood pooling was calculated. The lower LBNP pressure (11 mmHg) was chosen since change in calf volume below LBNP pressures of 10 mmHg may be dependent on other parameters than venous filling, not reflecting venous wall properties (23). The upper limit was chosen to avoid presyncope, frequently known to occur in women at LBNP levels over 45–50 mmHg (16, 17), and men with type 1 diabetes have shown signs of hemodynamic instability well below this level (8). After correcting for LBNP pressure transmission of roughly 80% (11), the studied venous transmural pressure interval was 9–36 mmHg, a range previously applied to both healthy subjects and men with type 1 diabetes (8, 17, 22–24).

### Venous Compliance

Calf venous compliance (ml/100 ml/mmHg) was measured by a modified version of the technique developed by Olsen and Lanne (11) and previously used (22, 23). In each subject, the LBNP-evoked blood pooling were plotted against the prevailing transmural pressure of 9, 18, and 36 mmHg (Figure 2A). The resulting blood pooling–pressure curve was non-linear, with larger volume changes (greater compliance) at lower transmural pressures as described by a quadratic regression equation:

(1) $$\Delta \;\mathrm{Calf}\;\mathrm{volume}=\beta_{0}+\beta_{1}\cdot \text{(transmural pressure)}+\beta_{2}\cdot {\mathrm{(transmural pressure)}}^{2}$$

β_0_ is the *y*-intercept, and β_1_ and β_2_ are characteristics of the slope of the volume–pressure curve. This equation showed an excellent mathematical fit to the measured data points (Figure 2A). Since compliance is altered with changes in pressure, no single value can characterize the slope of this relation. The first derivative of the volume–pressure curve [Compliance = β_1_ + (2 · β_2_ · transmural pressure)] was then calculated, creating a linear compliance–pressure curve (Figure 2B). Calf venous compliance was then calculated at various transmural pressures, in both the high and low ranges (Figure 2B). The use of the quadratic regression equation as a surrogate for true venous compliance is widely accepted, based on the work by Halliwill et al. (25).

In conjugation with the experiment described above, cardiovascular parameters were monitored, e.g., brachial blood pressure (Dinamap Pro 200, Critikon, Tampa, FL, USA) and forearm blood flow (FBF) [standard venous occlusion strain gage plethysmography (Hokanson EC-6, D. E. Hokanson, Bellevue, WA, USA)] (14).

### Statistical Evaluation

Values are expressed as means ± SE, unless stated otherwise. Group differences in blood pooling and calculated venous compliance were assessed using unpaired Student’s *t*-test. Bonferroni corrections were applied in subgroup analyses. One-way ANOVAs were applied to assess differences in continuous parameters (e.g., compliance at 20 mmHg) when studying subgroups as nominals (e.g., quartile groups based on HbA_1c5_). Two-way repeated measures ANOVAs were applied to assess group differences in blood pooling over transmural pressure of 9–36 mmHg and rate of blood pooling during the first 60 s of LBNP. Regression analyses were applied to assess correlations between continuous parameters of diabetes (e.g., duration of diabetes; see Table 1) and blood pooling as well as venous compliance. *P* < 0.05 was considered statistically significant.

## Results

### Baseline Characteristics

DW were slightly older than C (DW, 24.6 ± 0.8; C, 22.8 ± 0.3 years, and *P* < 0.05). No differences were seen in body height (DW, 1.66 ± 0.02; C, 1.70 ± 0.02 m) or body weight (DW, 66 ± 2; C, 62 ± 2 kg), with body mass index (BMI) slightly higher in DW, but well within normal range (DW, 23.7 ± 0.5; C, 21.2 ± 0.4 kg/m^2^, *P* < 0.01). Please see Table 2 for full cardiovascular characteristics at rest in both groups. DW presented with elevated resting heart rate (*P* < 0.0001) and increased pulse pressure (*P* < 0.001).

### Blood Pooling and Venous Compliance

**Figure 1:**
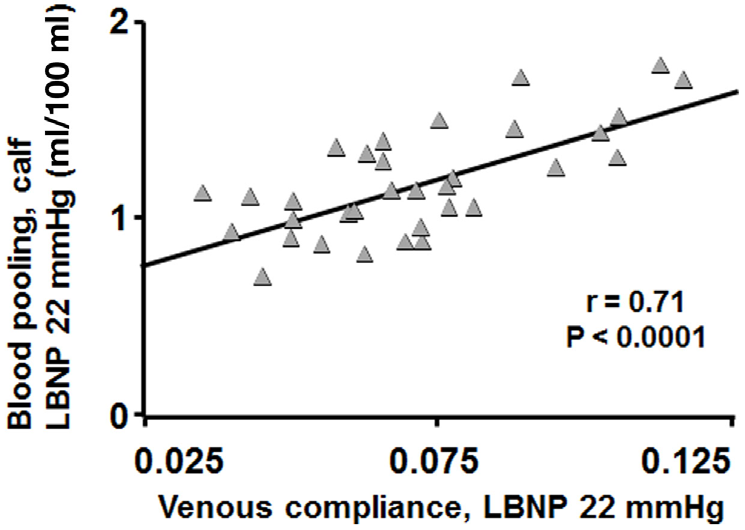
**Blood pooling in DW and C during LBNP of 22 mmHg plotted against their calculated venous compliance at concomitant evoked transmural pressure (gray triangles)**. Blood pooling correlated well with venous compliance (*r* = 0.71, *P* < 0.0001).

**Figure 2:**
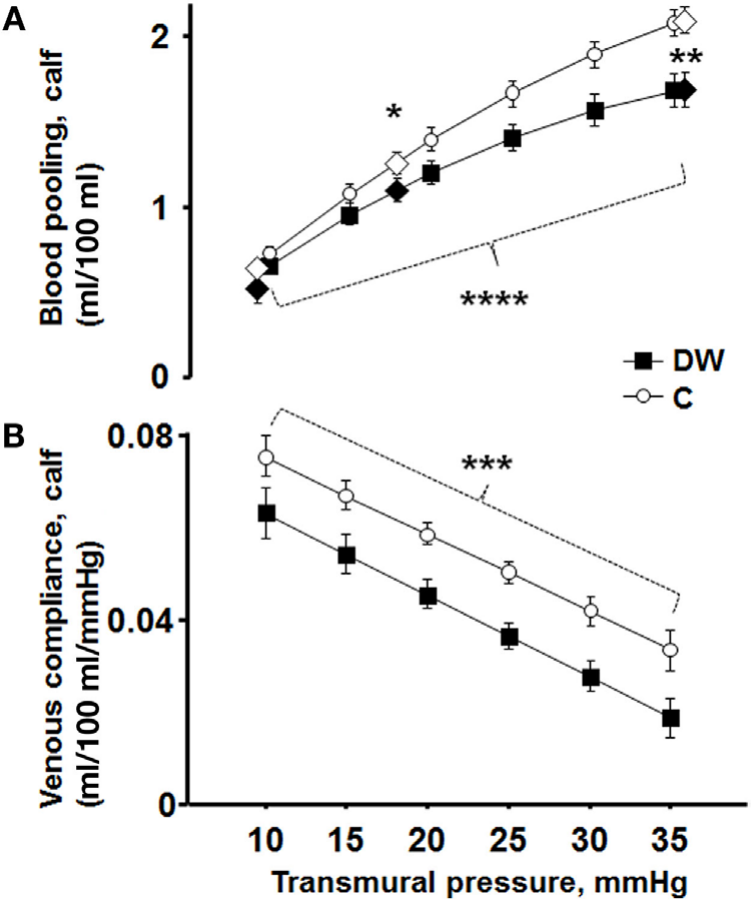
**(A)** Blood pooling at LBNP of 11, 22, and 44 mmHg (equivalent to transmural pressure of 9, 18, and 36 mmHg) in DW (black diamonds) and C (white diamonds). The from the quadratic regression equation calculated volume is depicted in two black lines marked every 5 mmHg (DW black boxes; C white circles). Blood pooling (ml/100 ml) was reduced in DW during LBNP of 22 mmHg (*P* < 0.05) and LBNP of 44 mmHg (*P* = 0.003). The shape of the line was more horizontal in DW with increased transmural pressure, i.e., blood pooling increased less with enhanced transmural pressure in DW (*P* < 0.0001). **(B)** Corresponding calf venous compliance in DW (black boxes) and C (white circles). Venous compliance (ml/100 ml/mmHg) was reduced in DW, both overall (*P* = 0.0007) as well as during calculated venous compliance (e.g., compliance at LBNP 22 mmHg, *P* = 0.002).

Figure 1 shows blood pooling evoked by LBNP of 22 mmHg plotted against its induced transmural pressure, with a clear correlation seen (*r* = 0.71, *P* < 0.0001). Figure 2A present calf blood pooling brought on by LBNP of 11, 22, and 44 mmHg (equivalent to transmural pressure of 9, 18, and 36 mmHg) as well as the line calculated by the quadratic regression equation during transmural pressure of 10–35 mmHg. Blood pooling (ml/100 ml) was reduced in DW during LBNP of 22 mmHg (DW 1.09 ± 0.07; C, 1.25 ± 0.06, *P* < 0.05) and LBNP of 44 mmHg (DW, 1.69 ± 0.10; C, 2.10 ± 0.08, *P* = 0.003) as well as overall reduced during the whole pressure range (10–35 mmHg, *P* < 0.05) and a more flat slope with increased transmural pressure in DW (*P* < 0.0001). The rate of calf volume increase (reflecting rate of blood pooling) during the first minute of LBNP 22 mmHg was similar in DW and C (*P* = 0.70). Figure 2B depicts the corresponding calf venous compliance in DW and C. Venous compliance (ml/100 ml/mmHg) was reduced in DW, both overall (*P* = 0.0007) as well as during calculated compliance between transmural pressure of 15–35 mmHg (e.g., compliance at 20 mmHg, DW, 0.046 ± 0.003; C, 0.059 ± 0.002, *P* = 0.002). The two separate components for venous compliance calculation were: β_1_, 0.081 ± 0.009 in DW and 0.092 ± 0.007 in C; β_2_, −0.00088 ± 0.00015 in DW and −0.00084 ± 0.00015 in C.

### HbA1c in Correlation with Blood Pooling and Venous Compliance

**Figure 3:**
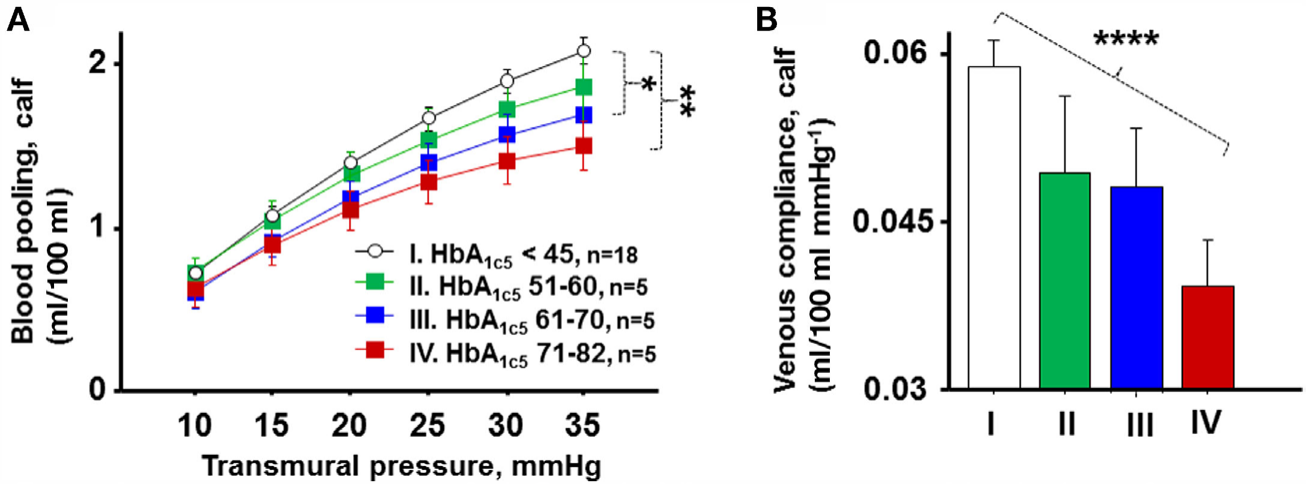
**(A)** Calf blood pooling divided into four groups based on HbA_1c5_ denoted normal (I, white circles, *n* = 18), mild (II, green boxes, *n* = 5), moderate (III, blue boxes, *n* = 5), and poor (IV, red boxes, *n* = 5). Blood pooling was progressively reduced in groups with increasing HbA_1c5_ (*P* = 0.005). Women with normal HbA_1c5_ had significantly greater blood pooling during LBNP of 44 mmHg than women with moderate and severe HbA_1c5_ (*P* = 0.02 and *P* = 0.001, respectively). **(B)** Corresponding mean venous compliance during transmural pressure of 10–35 mmHg in analogous groups (I–IV). A progressive reduction in venous compliance with increasing HbA_1c5_ throughout the studied pressure range of 10–35 mmHg was seen (*P* < 0.0001).

Figure 3A shows blood pooling in the calf divided into four groups based on mean HbA_1c_ the last 5 years (HbA_1c5_). Blood pooling was progressively reduced in groups with increasing HbA_1c5_ (*P* = 0.005). Women with normal HbA_1c5_ had significantly greater blood pooling during LBNP of 44 mmHg than women with moderate and severe HbA_1c5_ (*P* = 0.02 and *P* = 0.001, respectively), but no statistical differences were seen between the other three groups. Figure 3B presents corresponding venous compliance in analogous groups, with progressively reduced venous compliance with increasing HbA_1c5_ (*P* < 0.0001). A calculated venous compliance during transmural pressure of 20 mmHg was likewise progressively reduced in a similar fashion with increasing HbA_1c5_ (*P* = 0.007).

**Figure 4:**
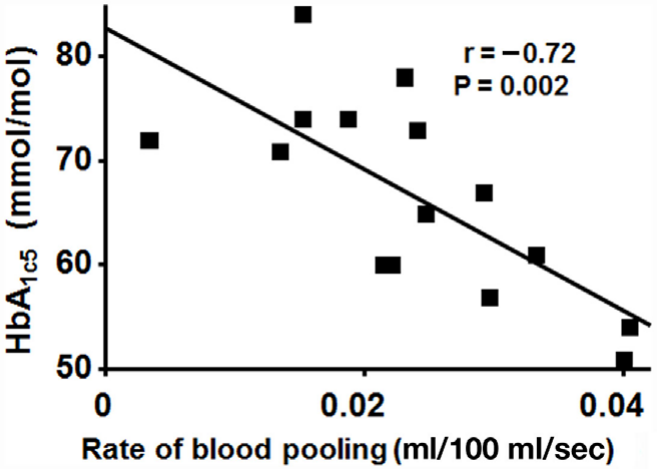
**Correlation between level of HbA_1c5_ and the mean rate of calf volume increase 30–60 s after LBNP initiation in DW**. A negative correlation seen (*r* = −0.72, *P* = 0.002), i.e., slower increase in blood volume with worse glycemic control.

Figure 4 shows the correlation between level of HbA_1c5_ and the rate of calf volume increase (ml/100 ml/sec) 30–60 s after LBNP initiation in DW, with a negative correlation seen (*r* = −0.72, *P* = 0.002), i.e., the higher the HbA_1c5_ value, the slower the blood pooling rate.

### Retinopathy in Correlation with Blood Pooling and Venous Compliance

**Figure 5:**
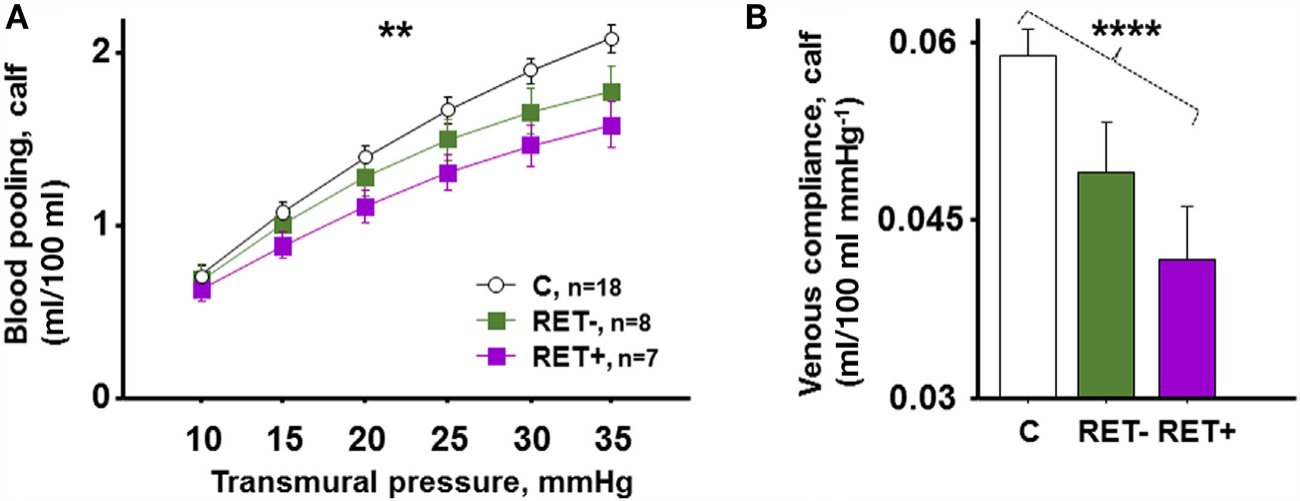
**(A)** LBNP-induced blood pooling in C (*n* = 18), RET− (DW with no signs of retinopathy, *n* = 8) and RET+ (DW with signs of retinopathy, *n* = 7). C pooled the greatest amount of blood, followed by RET− and RET+, with progressively reduced blood pooling seen in this order (*P* = 0.003). **(B)** Corresponding mean venous compliance during transmural pressure of 10–35 mmHg in identical groups, with gradually reduced venous compliance in C, RET−, and RET+ (*P* < 0.0001).

HbA_1c5_ tended to be increased in RET+ compared to RET− (*P* = 0.06). Figure 5A shows blood pooling evoked by LBNP depicted in C, RET−, and RET+. C seemingly pooled the greatest amount of blood, followed by RET− and RET+, with a progressive reduction in blood pooling (*P* = 0.003), a pattern also recurring at LBNP-induced blood pooling of 44 mmHg (*P* = 0.008). Figure 5B depicts corresponding venous compliance with gradually reduced venous compliance in C, RET−, and RET+ (*P* < 0.0001). A calculated venous compliance during transmural pressure of 20 mmHg was also correspondingly reduced (*P* = 0.003). No significantly detectable differences were seen between RET+ and RET− in blood pooling (*P* = 0.12) or venous compliance (*P* = 0.16).

### Blood Pooling, Venous Compliance, and Other Parameters of Disease Severity

Blood pooling and venous compliance were also compared with parameters stated in Table 1 (e.g., duration of diabetes and insulin dosage per kilogram body weight). Resting heart rate (here used as a surrogate marker for autonomic dysfunction) showed a weak, but significant, negative correlation with blood pooling at LBNP of 44 mmHg (*R* = −0.37, *P* = 0.03) and reduced venous compliance in all women studied (*P* = 0.04). Although some significant correlations fell out randomly between the parameters and blood pooling/venous compliance, no consistent patterns or correlations were seen.

## Discussion

The main findings in the present study were: blood pooling and venous compliance in the lower limb were reduced in women with type 1 diabetes. Worse glycemic control, but also the presence of microvascular disease and signs of autonomic dysfunction, were associated with further reduction in blood pooling and venous compliance.

### Hemodynamic Stability in Diabetes

Patients with diabetes are prone to hemodynamic instability and present with reduced tolerance to hypovolemia, most prominent in the short term during change from supine to erect body position and during anesthesia (1–4). The present study focuses mainly on these short-term hemodynamic effects of diabetes. LBNP is a widely used model for hypovolemic and orthostatic stress, mimicking the rapidly induced central hypovolemia when shifting body position from supine to erect, unloading the baroreceptors (7). Roughly, 70% of the total blood volume resides in the systemic veins, important for maintaining hemodynamic stability by serving for proper venous return to the right atrium (26). Compliant capacitance veins (great blood mobilization for each small reduction in venous pressure) is, therefore, a key factor in maintaining hemodynamic stability, e.g., during rapid changes in body position (13). However, it is a double-edged sword, where highly compliant veins could predispose for orthostatic hypotension as a result of a large shift of blood to the lower limbs during erect position, seen in the present study with greater blood pooling in women the greater the venous compliance (Figure 1).

### Venous Compliance and Blood Pooling in Diabetes

Venous compliance was reduced in DW (Figure 2B), in analogy with two previous studies on venous compliance and diabetes (9, 10). Venous compliance has not earlier been studied in diabetes in conjunction with orthostatic stress and its implications. We extend on previous findings by presenting reduced blood pooling in DW (Figure 2A), which could as such serve as a protective mechanism against orthostatic hypotension in DW (smaller decrease in central blood volume), counterbalancing the occurrence of autonomous dysfunction (2, 4, 27).

Men with type 1 diabetes present with reduced blood pooling only when microvascular disease was present (8, 28). Venous compliance was, however, not calculated in these studies. The role of type 1 diabetes in stiffening of elastic arteries (compliance reduction) also seem more prominent in women with type 1 diabetes than men (15). Young women also have lower tolerance to orthostatic stress than men (16), collectively emphasizing the importance to study blood pooling and venous compliance in women with type 1 diabetes.

The reduced venous compliance could in part explain the seen reduction in hypovolemic stress-induced compensatory mobilization of capacitance blood in diabetes (8, 14). Both lower limb blood pooling and compensatory mobilization of capacitance blood has been linked with diabetes severity (8, 14). With this in mind, a sub study was conducted, comparing blood pooling and venous compliance with various markers of diabetes severity (Table 1). In the commencing sub studies, the number of women with diabetes in each subgroup were small (*n* = 5), and as such, the results must be interpreted with caution. Nevertheless, significant findings in line with the hypothesis of the study and the pathophysiology of diabetes were found.

### Levels of HbA1c, Blood Glucose, and Insulin Dosage

Both blood pooling and venous compliance decreased with increasing level of HbA_1c5_ (Figures 3A,B). Prolonged poor glycemic control was also associated with slower pooling of blood in the lower limb (Figure 4). The slower blood pooling could be an independent contributing factor to hemodynamic instability seen in diabetes during orthostatic stress (4, 17) It is well known that poor glycemic control over time increases glycation of proteins, undergoing complex reactions to become irreversibly cross-linked, termed advanced glycation end products (AGEs). AGEs accumulate on collagen and elastin in the vessel wall and are likely involved in the increase in vascular stiffness seen in diabetes (29–31). It seem plausible that poor glycemic control and increased amount of AGEs contribute to reduce compliance also on the venous side. In analogy, mean level of HbA_1c_ in the 5 years preceding the study showed a stronger negative correlation with venous compliance than level of HbA_1c_ at the time of the study, further corroborated with the lack of association between levels of blood glucose during experiments and venous compliance. Insulin is known to affect various parts of the cardiovascular system, but we found no correlation between daily insulin dose or insulin dosage per kilogram body weight and venous compliance. Taken together, it seems that the reduction in venous compliance in diabetes is caused by long-term effects of diabetes rather than short-term differences in blood glucose or insulin.

### Presence of Microvascular Disease and Autonomic Dysfunction

Clinical examination revealed evidence of background retinopathy in seven DW, with four of these categorized as slight or minimal and none with proliferative retinopathy. As such, none of the DW had more than mild microvascular engagement, and no one presented with clinical signs of neuropathy or nephropathy (Table 1). Both reduced venous compliance and blood pooling were seen when dividing the women into groups of healthy, RET− and RET+ (Figure 5; *P* < 0.0001). However, no statistically significant difference was seen between RET− and RET+ when comparing venous compliance (*P* = 0.16) and blood pooling (*P* = 0.12). This is in contrast to a previous study in men with diabetes, presenting reduced blood pooling with presence of microvascular disease (8). However, the male diabetics presented with more aggravated microvascular disease as well as evidence of neuropathy and/or nephropathy, possibly explaining the differences found (8).

### Other Signs of Diabetes Severity

DW presented with increased heart rate at rest (Table 2), a sign of autonomic dysfunction (27). We saw no correlation between resting heart rate and venous compliance and blood pooling within DW alone. When pooling both groups together, a weak negative association was seen, i.e., the greater the heart rate, the lower the venous compliance (see Section “Results”). The finding could be a result of group differences between healthy controls and DW rather than influence of autonomic dysfunction on venous compliance. However, compensatory mobilization of venous capacitance blood during hypovolemic stress (dependent on high venous compliance) is clearly associated with autonomic dysfunction (14). This discrepancy could be explained by physiological differences, where venous compliance in the present study is measured within the transmural pressures range of 9–36 mmHg and the decrease in transmural pressures in the venous section triggering compensatory mobilization of blood is well below 5 mmHg (24). As such, it is likely that the autonomic dysfunction present in DW more prominently affects compliance in the low pressure area in early stages of the disease.

Cardiovascular autonomic neuropathy is common in diabetes patients and increases with duration of disease, although difficult to diagnose in its early stages (2, 32). No correlation was seen between venous compliance and age at onset of diabetes or duration of the disease. Furthermore, blood pooling and venous compliance were nowhere near associated with glomerular filtration rate (GFR), cholesterol level, or detectable microalbuminuria (Table 1).

The diabetes care in Linköping is of high international standard, and the participating young women with type 1 diabetes presented with overall good glycemic control and were free of aggravated microvascular disease (Table 1). In this setting, any possible association between diabetes severity and studied venous compliance and blood pooling in young women will be harder to detect. Further studies including middle-aged men and women with type 1 diabetes is therefore warranted.

### Limitations of the Study

Calf venous compliance was measured in accordance with previously published work from our laboratory [e.g., Ref (22, 23)], by accurately decreasing extravascular tissue pressure (LBNP) to generate transmural pressure differences over the venous wall rather than increasing intravascular pressure with a thigh cuff (25). This approach has advantages in that it allows for easy adjustment of the prominent calf net fluid filtration present conjoined with blood pooling during both LBNP and cuff technique. We have worked with both techniques and feel that they both are reliable in measuring venous compliance.

## Conclusion

Venous compliance was reduced in women with type 1 diabetes. Blood pooling at transmural pressures relevant to upright posture was also reduced in women with type 1 diabetes. Worse glycemic control over time was correlated with further aggravated reduction in venous compliance. These data suggest pathophysiological incorporation of advanced glycemic end products in the vessel wall contributing to the reduction in venous compliance. A less compliant venous system could contribute to the hemodynamic instability associated with diabetes.

## Author Contributions

ML was responsible for the study design, performed the experiments, compiled and interpreted the data, and drafted the manuscript.

## Conflict of Interest Statement

The research was conducted in the absence of any commercial or financial relationships that could be construed as a potential conflicts of interest. No other conflict of interest exist.
